# Role of wavelength in photocarrier absorption and plasma formation threshold under excitation of dielectrics by high-intensity laser field tunable from visible to mid-IR

**DOI:** 10.1038/s41598-020-70862-w

**Published:** 2020-08-19

**Authors:** Ekaterina Migal, Evgenii Mareev, Evgeniya Smetanina, Guillaume Duchateau, Fedor Potemkin

**Affiliations:** 1 Faculty of Physics and International Laser Center, M.V. Lomonosov Moscow State University, Moscow, Russia; 2 Faculty of Physics, M.V. Lomonosov Moscow State University, Moscow, Russia; 3 Centre Lasers Intenses et Applications, UMR5107, University of Bordeaux-CNRS-CEA, 33405 Talence, France

**Keywords:** Nonlinear optics, Ultrafast photonics, Electronic properties and materials, Laser-produced plasmas

## Abstract

The development of high power mid-IR laser applications requires a study on laser induced damage threshold (LIDT) in the mid-IR. In this paper we have measured the wavelength dependence of the plasma formation threshold (PFT) that is a LIDT precursor. In order to interpret the observed trends numerically, a model describing the laser induced electron dynamics, based on multiple rate equations, has been developed. We show both theoretically and experimentally that PFT at mid-IR wavelengths is controlled by a transition from weak- to strong-field regime of free carrier absorption. In the case of MgF$_2$ this transition occurs around 3–4 $\upmu$m corresponding to the region of the lowermost PFT. The region of the uppermost PFT is reached around 1 $\upmu$m and is governed by an interplay of photoionization and weak-field free carrier absorption which manifests itself in both MgF$_2$ and SiO$_2$. The PFT observed in considered materials exhibits a universal dependence on the excitation wavelength in dielectrics. Thus, the presented results pave the route towards efficient and controllable laser-induced material modifications and should be of direct interest to laser researchers and application engineers for prevention of laser-induced damage of optical components in high-intensity mid-IR laser systems.

## Introduction

Recent advances in the development of ultra-short laser sources in mid- and far-infrared (IR) regions^1–8^ lead to a vast number of new applications. However, a primary technological challenge still relevant for researches is the manufacturing of optical components with high laser-induced damage threshold and desired properties (for instance, high transparency or reflectivity in a wide wavelength range, group delay, etc.). This implies a need for experimental investigation of material damage threshold in the IR spectral region^9,10^. Thin-films made of multi-layer stacks of low (fluorides, SiO$_2$) and high (silicon, germanium) refractive index materials are a key component of high-performance coatings in the mid-IR. Fluorides are also popular host matrices for mid-IR solid-state^11^ and fiber lasers^12^. The knowledge on damage threshold is also important for a new fascinating mid-IR application of dielectric laser accelerators (DLAs)^13,14^. In the mid-IR spectral region, promising materials for on-chip DLA technology are dielectric materials, which have a higher material damage threshold compared to silicon^14,15^. Well-developed applications such as ultrafast laser materials processing^16^ and direct laser writing^17^ may also have advantages in switching from visible and near-IR to mid-IR. A promising way for controlling the energy deposition inside transparent dielectric materials is to use a two-color ultrafast excitation^18–24^. In this scheme a long wavelength heating pulse is more advantageous due to favorable scaling of avalanche ionization (AI) rate and free electron absorption. However, a lack of knowledge of the laser-induced damage threshold (LIDT) dependence on the wavelength prevents the optimization of such a concept.

The complexity of predicting the LIDT behavior with wavelength is associated with a wavelength dependence of both photoionization and impact ionization rates. The rate of multi-photon ionization (MPI) decreases with wavelength, while tunnel ionization (TI) exhibits no wavelength dependence^25^. Electrons in the conduction band produced by photoionization can be further heated by laser pulse through direct or phonon assisted absorption. Effective collision frequency can be found in two cases of a weak and strong field, transition between which is determined by relation between the average kinetic energy of an oscillating electron and the photon energy^26^. When the cycle-averaged quiver energy of a free electron becomes much higher than the photon energy, high order processes (such as simultaneous absorption of several photons for one collisional event) may provide significant contribution. These high order processes drastically change electron heating rate and influence the total energy density in the electronic subsystem. In the visible and near-IR regions, transition between the regimes is reached for intensities in excess of 100 TW/cm$^2$^27^. However, this transition occurs at much lower intensity in the mid-IR due to much higher ponderomotive energy. Therefore, all the mentioned processes have to be included to account for the observed LIDT behavior with respect to the wavelength.

Despite the presence of multiple theoretical models, the entire range of wavelengths has not been fully compared with their predictions. LIDT from the visible to mid-IR has been firstly addressed in early works of Manenkov’s group and reviewed in^28^. Due to quite low intensity of nanosecond pulses only regimes of MPI and seeded AI at 0.69, 1.06 and 2.76 $\upmu$m were observed. At longer wavelengths (10.6 $\upmu$m) avalanche was deterred due to the low seeding, while no transition between the regimes (of weak and strong field) has been captured. Simanovskii et al.^29^ have revealed that depending on the material bandgap, the TI alone or TI followed by AI may lead to a damage by mid-IR picosecond pulses (4.7–7.8 $\upmu$m). Other works which were carried out with femtosecond driving pulses were, unfortunately, limited to the visible and near-IR wavelengths leaving the ionization mechanisms at longer wavelengths experimentally unstudied. An increase of the LIDT in wide-gap materials with the incident pulse wavelength from visible to near-IR was confirmed by several studies at femtosecond time scale^30–32^. It was associated with the decrease of the MPI rate with the wavelength, that leads to a reduction in the seed electrons for subsequent laser heating and impact ionization. In the range of 1200–2000 nm, the nonlinear absorption is shown to be independent on the wavelength for SiO$_2$^30,33^ and slightly decreases in CaF$_2$^30^. Therefore, in order to better understand the mechanisms of ionization and laser-induced heating of the conduction-band electrons in the wavelength range from the visible to mid-IR, additional experimental studies and further theoretical modeling incorporating all the mentioned above processes is required.

In this work, we present experimental and theoretical results aiming at uncovering new features of femtosecond laser excitation of dielectric solids with mid-IR high-intensity laser pulses. We experimentally investigate the influence of the incident pulse wavelength on the plasma formation under femtosecond excitation of dielectric materials (SiO$_2$, MgF$_2$) in the visible, near-IR and mid-IR spectral regions. The plasma formation threshold (PFT) as a precursor of LIDT is determined by the nonlinear transmission measurements which provide higher precision than the LIDT measurements. We use numerical simulations of the electron plasma formation and dynamics by means of Multiple Rate Equations (MRE) to interpret experimental data, revealing an interplay of the ionization mechanisms in the chosen range of incident pulse wavelengths. We report on a non-monotonic behaviour of the measured PFT that reaches its maximal values in near-IR wavelength range and minimal values around 2–4 $\upmu$m.

## Results and discussion

**Figure 1:**
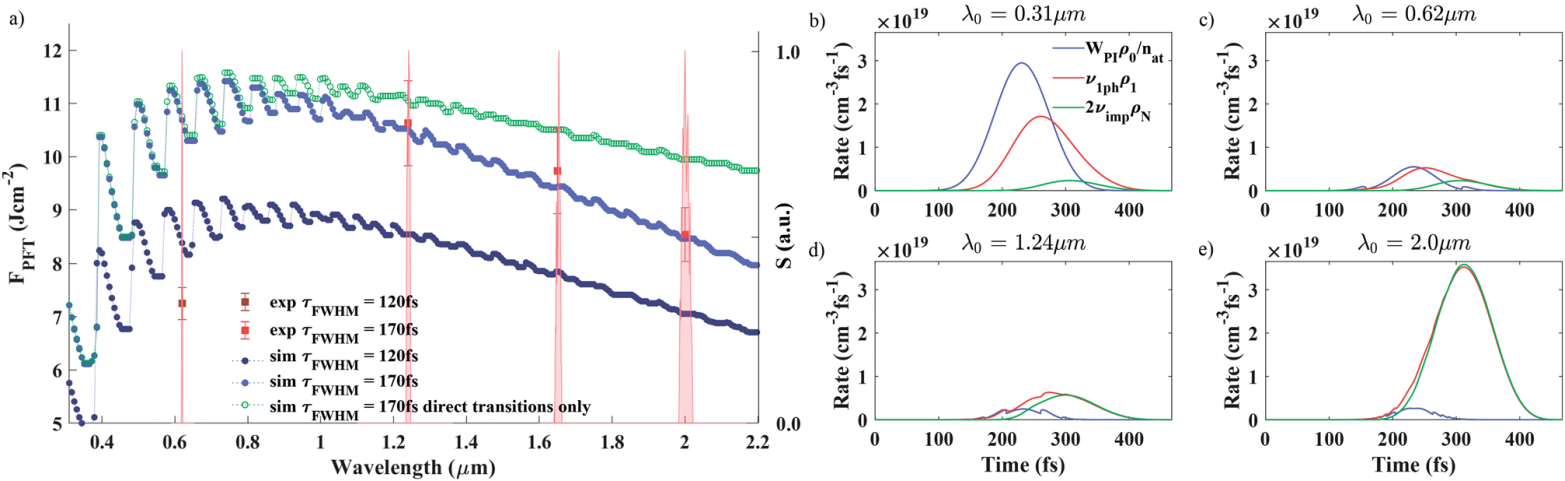
(**a**) PFT of SiO$_2$ for various wavelengths. Experimental results are presented by red squares with error bars. Simulations are presented by circles and dotted lines as a guide for the eye. Light and dark blue circles correspond to the values of $W_{1ph}^{dr,idr}$ and 120-fs and 170-fs pulse duration, respectively. Green circles correspond to the simulation with electron-phonon-photon interactions switched off, i.e. $W_{1ph}^{idr} = 0$ and 170-fs pulse duration. Experimental spectra are also shown by light red. (**b**–**e**) Keldysh ionization rate $W_{PI}\rho _0/n_{at}$ (blue), one-photon transition rate from the 1st to the 2nd CB level $\nu _{1ph}\rho _1$ (red) and impact ionization rate $2\nu _{imp}\rho _N$ (green) as a function of time for (**b**) 0.31 $\upmu$m, (**c**) 0.62 $\upmu$m, (**d**) 1.24 $\upmu$m and (**e**) 2.0 $\upmu$m at the fixed fluence F = 8.4 J cm$^{-2}$ that is close to $F_{PFT}$ value at 0.31 $\upmu$m in SiO$_2$. Pulse duration is $\tau _{FWHM} = 170fs$.

**Figure 2:**
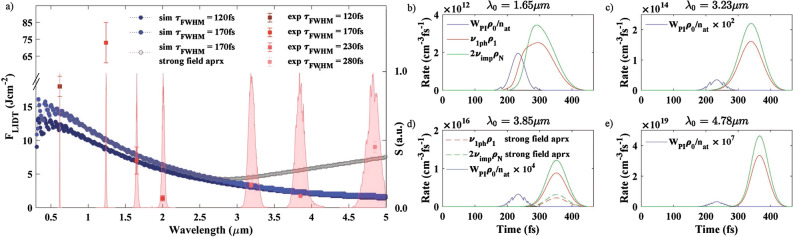
(**a**) PFT of the $MgF_{2}$ for different wavelengths. Experimental results are presented by red squares with error bars. The PFTs at 3.23 $\upmu$m and 3.85 $\upmu$m have the errors ± 0.4 J cm$^{-2}$ and ±0.2 J cm$^{-2}$, respectively. Simulations are presented by circles and dotted lines as a guide for the eye. Filled circles correspond to the values of $W_{1ph}^{dr,idr}$. Gray empty circles correspond to the simulation with strong field approximation for $\widetilde{W}_{1ph}^{sf}$. Pulse duration is indicated on the graph. Experimental spectra are also shown by light red. (**b**–**e**) Keldysh ionization rate $W_{PI}\rho _0/n_{at}$ (blue), one-photon transition rate from the 1st to the 2nd CB level $\nu _{1ph}\rho _1$ (red) and impact ionization rate $2\nu _{imp}\rho _N$ (green) as a function of time along the laser pulse for (**b**) 1.65 $\upmu$m, (**c**) 3.23 $\upmu$m, (**d**) 3.85 $\upmu$m and (**e**) 4.78 $\upmu$m at the fixed fluence F = 2.3 J cm$^{-2}$ that is close to $F_{PFT}$ value at 3.85 $\upmu$m in MgF$_2$. Pulse duration is $\tau _{FWHM} = 170fs$. Note that the scales are different in the different sections of the figure. The Keldysh rate $W_{PI}\rho _0/n_{at}$ is multiplied by the factors indicted to be distinguished from zero. Dashed curves correspond to the strong field approximation included.

Figures 1(a) and 2(a) show the PFT dependence on the driving wavelength for SiO$_2$ and MgF$_2$ (experimental values are shown by squares with error bars). Only visible and near-IR spectral regions are presented in the case of SiO$_2$ due to appearance of linear absorption beyond 2.6 $\upmu$m. Experimentally the PFT was determined by appearance of the nonlinear absorption. The value of 5$\%$ absorption was chosen for confident registration due to 3$\%$ root-mean-square stability of the driving source (see Methods for more details). Note that the PFT (unlike LIDT) is almost unaffected by the propagation effects since plasma is generated in the volume smaller than the beam waist^34^. In the numerical simulations, the PFT is assumed to be reached if the total electron density at the end of the laser pulse $\rho _{\mathrm{e}}(t=\tau _0)$ is equal to $10^{-1}\times n_{at}$ that are close to the plasma density threshold values used in other works^27,35,36^. The corresponding fluence of the laser pulse is called $F_{PFT}$ and is calculated for all wavelengths in the considered range. This PFT criterion has been compared with both the criteria of reaching the critical plasma density $\rho _{cr}$, and reaching the LIDT defined as a threshold value of the energy density in the electronic subsystem^31^. Due to the wavelength dependence of the critical plasma density $\rho _{cr}$, the corresponding threshold exhibits a monotonic decrease (besides jumps due to a change in the multiphoton order) with the wavelength in the entire range from UV to mid-IR (results not shown), that departs from the experimental observations. The LIDT criterion leads to a behaviour with respect to the wavelength similar to the PFT criterion $10^{-1}\times n_{at}$, that is in a good agreement with the experimental observations^32^ and is a further evidence that the PFT (reach of a given free electron density) is the precursor of LIDT. For both samples experimentally and numerically non-monotonic behavior of the PFT was observed. Firstly, the PFT increases with wavelength from visible to near-IR. However, at longer wavelengths the PFT tends to decrease, that is particularly pronounced in the case of MgF$_2$. Finally, the observed PFT in MgF$_2$ exhibit an increase around 5 $\upmu$m.

It is worth mentioning that generally irreversible damage of the material occurs when energy deposition into material reaches a value sufficient for material melting or phase transition to take place. For instance, in SiO$_2$ the LIDT is typically 1.2–1.5 times higher than PFT, since for the material damage absorption should be at the level of 20–50% (which was determined experimentally, see Fig. 3). However, measurement of LIDT values is strongly influenced by the glass sample quality, measuring methods and even focusing conditions of the laser beam^37–40^ since tiny modification of the material is hard to capture especially in the bulk. Considering only the beam waist impact, the measured LIDT in SiO$_2$ grows from 4 to 26 J cm$^{-2}$ with the beam waist decrease from 14 to 1 $\upmu$m^40^. That makes a direct comparison of the LIDT measurements presented in various sources practically not feasible. Thus, our goal is to evaluate generic dependencies of the LIDT values on the laser pulse wavelength rather than to compare directly the LIDT values from various references. Since the PFT can be measured with higher precision using nonlinear transmission or third harmonic generation methods, we show here only the PFT experimental dependencies. However, the overall behavior of the PFT and LIDT with wavelength are close^32^.

We simulate numerically the PFT at various incident laser pulse wavelengths covering the experimental range. As can be seen from Figs. 1(a) and 2(a), the dependence of the PFT on the wavelength appears to have strong oscillations in the visible and near IR spectral region. This behaviour is provided by Keldysh ionization rate and corresponds to the change of the multiphoton order with wavelength. Since in the mid-IR region tunneling ionization prevails in wide gap dielectrics, oscillations become less resolved and are smoothed out.

We start the analysis with the SiO$_2$ case, first considering an overview of the related LIDT measurements given in the literature since the overall behavior of the PFT and LIDT with wavelength are close^32^. The LIDT measurements in SiO$_2$ for a 100-fs laser pulse with wavelengths from 310 to 1030 nm show an oscillating behaviour of the LIDT with an overall increase of its value from 1 to 2.2–2.5 J cm$^{-2}$^31^. Measurements in a wider wavelengths range, from 267 to 1550 nm, show that the LIDT grows from 0.9 J cm$^{-2}$ at 276 nm up to above 3 J cm$^{-2}$ at 1300 nm, and then slowly drops down to 2.9–3 J cm$^{-2}$ at 1550 nm^30^. The measured PFT in this work has similar behaviour. The PFT reaches its maximal value (over the measured wavelengths) at 1240 nm and slowly decreases from 1240 to 2000 nm (Fig. 1a).

The highest PFT fluence in our simulations is reached for wavelengths from 0.6 to 0.9 $\upmu$m for both 120-fs and 170-fs laser pulses (Fig. 1(a), dark and light blue circles, correspondingly). The PFT decrease along both the high- and low-photon-energy sides of the spectral region is in a good agreement with experimental data.

The presented PFT behaviour can be explained as follows. First of all, we indicate the dependencies of the considered electron transitions on the incident photon energy: (1) The electron transitions from the VB to the first CB level are governed by the Keldysh photoionization rate which increases with the photon energy (besides changes in the multiphoton order due to variations of the ponderomotive energy); (2) The CB electron transitions are governed by $W^{dir}_{1ph}$ and $W^{idr}_{1ph}$ that decrease proportionally to $\omega _0^{-2}$ and $\omega _0^{-4}$, respectively; (3) The number of CB levels decreases with the photon energy (because the highest state has an energy of the order of the bandgap). To illustrate how these processes contribute to the electron density increase in the CB, we plot the Keldysh ionization rate $W_{PI}\rho _0/n_{at}$, one-photon transition rate from the 1st CB level to the 2nd CB level $\nu _{1ph}\rho _1$, and impact ionization rate $2\nu _{imp}\rho _N$ [Eq. (4)] as a function of time for 0.31 $\upmu$m, 0.62 $\upmu$m, 1.24 $\upmu$m and 2.0 $\upmu$m (Fig. 1b–e). For the considered wavelengths, the pulse fluence *F* is 8.4 J cm$^{-2}$, that is close to $F_{PFT}$ value at 0.31 $\upmu$m (Fig. 1a). It is worth mentioning that the strong field conditions are not reached in these ranges of the laser pulse wavelengths, duration and fluence $F_{PFT}$ in SiO$_{2}$.

In the UV spectral region the major contribution to the produced free electron density is provided by the photoionization process. The impact ionization is weak and does not contribute significantly (Fig. 1(b), $\lambda _0$ = 0.31 $\upmu$m). The photoionization rate decreases with the wavelength and the electron transitions in the CB are not sufficiently strong to develop an avalanche in the visible spectral range (Fig. 1c), $\lambda _0$ = 0.62 $\upmu$m). Further wavelength increase up to 1.24 $\upmu$m leads to further drop of the photoionization rate, while the electron transitions in the CB become sufficiently strong to develop impact ionization with peak rate above the photoionization one (Fig. 1d), $\lambda _0$ = 1.24 $\upmu$m). In the mid-IR region (Fig. 1e), $\lambda _0$ = 2 $\upmu$m) the photoionization rate keeps the same value while the electron transition in the CB drive a well-developed avalanche process with peak rate about one order of magnitude larger than the photoionization rate. Thus, the wavelength region of the highest PFT (0.59–0.82 $\upmu$m on Fig. 1a) appears due to the photoionization process, being already significantly weaker than in the UV region, and the electron transitions in the CB being not yet sufficiently strong to develop an avalanche. In the mid-IR the photoionization rate runs in the tunneling regime and the impact ionization mainly contributes to the growth of the free electron density (Fig. 1d–e, $\lambda _0$ = 1.24 $\upmu$m and 2.0 $\upmu$m). Note that by introducing an initial free electron density up to $10^{-6} \times n_{at}$ ($\rho _{1}(t=0) = 10^{-6}\times n_{at}$) does not change the PFT behaviour with the wavelength (results not shown), that indicates low levels of impurities in the glass sample where the PFT evolution in the mid-IR is driven by intrinsic processes including electron–photon and electron–photon–phonon processes in the CB.

In order to figure out the origin of the presented PFT behaviour, we performed simulations of PFT that accounts only for direct transitions between the CB levels and thus, excludes the indirect phonon-assisted transitions $W_{1ph}^{idr}=0$ (Fig. 1a, green circles). The PFT including only direct transitions in the CB is similar to the PFT obtained by the full modeling in the high-photon-energy spectral region (Fig. 1a, green and light blue circles, correspondingly). In the mid-IR spectral region, the PFT obtained by the modeling including only direct CB transitions is larger than the PFT given by the full modeling. In the mid-IR the variations of PFT with the wavelength are more pronounced with the indirect transitions due to the stronger dependence of $W_{1ph}^{idr}$ on the photon energy, that corresponds to the experimental observations. An increase of $W_{1ph}^{dr}$ in the modeling with direct CB transitions leads to lower PFT values, but the behaviour presented here will be kept, i.e. the decrease of the PFT with the wavelength in the mid-IR is still slower than the experimentally observed one (checked numerically, results not shown). Thus, in the visible spectral region, the direct electron transitions play a major role, while in the mid-IR, the indirect electron transitions significantly contribute in the energy accumulation process by the electronic subsystem of SiO$_2$.

In the following we switch to the MgF$_2$ sample, where the measured PFT decreases with wavelength from near-IR to mid-IR region. A local PFT minimum is reached around 3–3.85 $\upmu$m, followed by a further PFT increase with wavelength up to 4.78 $\upmu$m. An outstandingly large PFT at 1.24 $\upmu$m has been detected in extensive measurements with multiple cross-checks of the incident pulse parameters. An overview of the LIDT measurements given in the literature shows that for long pulses (1 ps) the LIDT measured in MgF$_2$ has close values in mid-IR (4.7 $\upmu$m) and near-IR (0.8 $\upmu$m): ~ 5.4 J cm^−2^ and ~ 4 J cm^−2^, correspondingly^29^. For shorter laser pulses (~ 500 fs), the measured LIDT are as follows: ~ 4.2 J cm^−2^ at 1030 nm^41^ and ~ 3.9 J cm^−2^ at 800 nm^42,43^. Due to a lack of broad data on the MgF$_2$ LIDT measurements, we also analyse the available LIDT in CaF$_2$ that is expected to exhibit the wavelength dependence of LIDT similar to it in MgF$_2$ because of the similar band gap values and strong electron–photon–phonon coupling in these materials^44^. In CaF$_2$, the LIDT for a 100-fs laser pulse with wavelengths from 400 to 1030 nm reaches its maximum value about 4 J cm$^{-2}$ at 800 nm^31^. Measurements in a wider wavelength range, from ~  400 to ~ 1900 nm, show that the LIDT grows from ~ 1.2 J cm$^{-2}$ at ~ 400 nm up to 3 J cm$^{-2}$ at 1400 nm and then drops down to ~2.3 J cm^−2^ at $\sim$1900 nm^30^. To conclude, the measurements in CaF$_2$ show the existence of a LIDT maximum in the near IR, the exact position of which varies with experimental conditions and laser parameters. These statement is also supported by the MgF$_2$ PFT measurements presented in the current work (Fig. 2a).

Our simulations of PFT revealed almost the same tendencies in visible and near-IR spectral regions in the case of MgF$_2$ (compared to SiO$_2$) (Fig. 2a). Strong field ionization is the dominant mechanism for excitation of CB electron in the case of high photon energies. However, in the near- and mid-IR (1.5–4 $\upmu$m) direct and, especially, indirect transitions provide electron density growth in the CB. Note that the present model does not account for the measured PFT at 1.24 $\upmu$m. We currently do not have any explanation of the observed difference. An improved theoretical approach is required for the spectral region around 1 $\upmu$m that lays in-between spectral regions that can successfully be described by means of quantum mechanical or classical approaches^45^. It is worth mentioning that the model only includes a few adjustable parameters whereas a very large wavelength region is considered, which strongly constrain the model and makes reasonable the present interpretations of experimental data.

To explain the PFT behaviour in the mid-IR, we plot the Keldysh photoionization rate $W_{PI}\rho _0/n_{at}$, one-photon transition rate from the 1st CB level to the 2nd CB level $\nu _{1ph}\rho _1$ and impact ionization rate $2\nu _{imp}\rho _N$ [Eq. (4)] as a function of time for 1.65 $\upmu$m, 3.23 $\upmu$m, 3.85 $\upmu$m and 4.78 $\upmu$m (Fig. 2b–e). The pulse duration is set to $\tau _{FWHM} = 170fs$ (Fig. 2a, filled light blue circles). For the considered wavelengths, the pulse fluence is 2.3 J cm$^{-2}$, that is close to $F_{PFT}$ value at 3.85 $\upmu$m (Fig. 2a). The peak value of the photoionization rate is almost the same for wavelengths 3.23 $\upmu$m, 3.85 $\upmu$m and 4.78 $\upmu$m, and is several times lower than for 1.65 $\upmu$m. The ionization-induced seed electrons at the lowest CB level appear with the same probabilities independently on the wavelength in the tunneling regime ($W_{PI}\rho _0/n_{at}$, blue curves for wavelengths 3.23 $\upmu$m, 3.85 $\upmu$m and 4.78 $\upmu$m Fig. 2c–e). In opposite, the one-photon absorption probabilities calculated in the weak field approximation are rapidly growing with the wavelength and start the avalanche process (red and green solid curves in Fig. 2b–e). That leads to the drop of the PFT calculated under the weak-field approximation when the wavelength increases (Fig. 2a, light blue filled circles). Taking into account the invalidity of the weak field approximation along the high-intensity laser pulse in the mid-IR, Eq. (7) is used when the condition for the strong-field approximation is fulfilled. The simulated dependence of the PFT on the wavelength exhibits an increase in that case (Fig. 2a, gray empty circles). In strong field approximation the effective electron absorption rate in the CB decreases with the increase of the electric field [Eq. (7)] that leads to a weaker avalanche development in the laser pulse tail (red and green dashed curves for 3.85 $\upmu$m in Fig. 2c) and corresponding increase of the calculated PFT (Fig. 2a, gray empty circles).

Thus, we are able to reproduce the experimentally observed PFT in a wide range of wavelengths from UV to mid-IR in MgF$_2$. We observe a departure of the theoretical modeling form the experimental observations in the region from 3 to 4 $\upmu$m. The results can be improved by adding initial seed electrons with a density of $10^{-8}\times n_{at} - 10^{-7}\times n_{at}$. They lead to the drop of the PFT at the wavelengths from 3 to 4 $\upmu$m but do not change the PFT increase at around 4–5 $\upmu$m (results not shown).

## Conclusion

We have measured the PFT for a set of wavelengths in the visible, near-IR and mid-IR spectral ranges for SiO$_2$ and MgF$_2$ dielectric materials. A non-monotonic behavior of the PFT has been observed. The main trends of the PFT evolution with respect to the wavelength is described by MRE model accounting for the laser induced electron dynamics in the material. This modeling reveals the interplay of the ionization mechanisms and allows us to shed light on the main mechanisms responsible for the PFT value in the considered spectral range. Decrease of the PFT at high photon energies is associated with the photoionization process, while indirect phonon-assisted transitions in the CB are responsible for its drop at lower photon energies leading to a well-developed avalanche ionization regime. We also revealed the influence of crystal impurities which may provide additional seed electrons. Efficient heating of these extra electrons is expected to be the origin of low PFT around 3 $\upmu$m in MgF$_2$, where otherwise electron heating is not efficient due to an absent of sufficient amount of photoionized free electrons. We have shown that the PFT in MgF$_2$ increases with the wavelength from 3.85 to 4.78 $\upmu$m due to transition from weak to strong regime and coming into play higher order process (such as multiphoton inverse Breamsstrahlung). Thus, spectral regions in MgF$_2$ and SiO$_2$ are found where local maximum and minimum of the PFT are reached. These findings provide new insights into interaction mechanisms of high-intensity mid-IR laser fields with dielectrics and open new opportunities for optimization of high-LIDT mid-IR components, as well as, extension of matter manipulation techniques by high-intensity laser pulses in the wide spectral range.

## Methods

**Figure 3:**
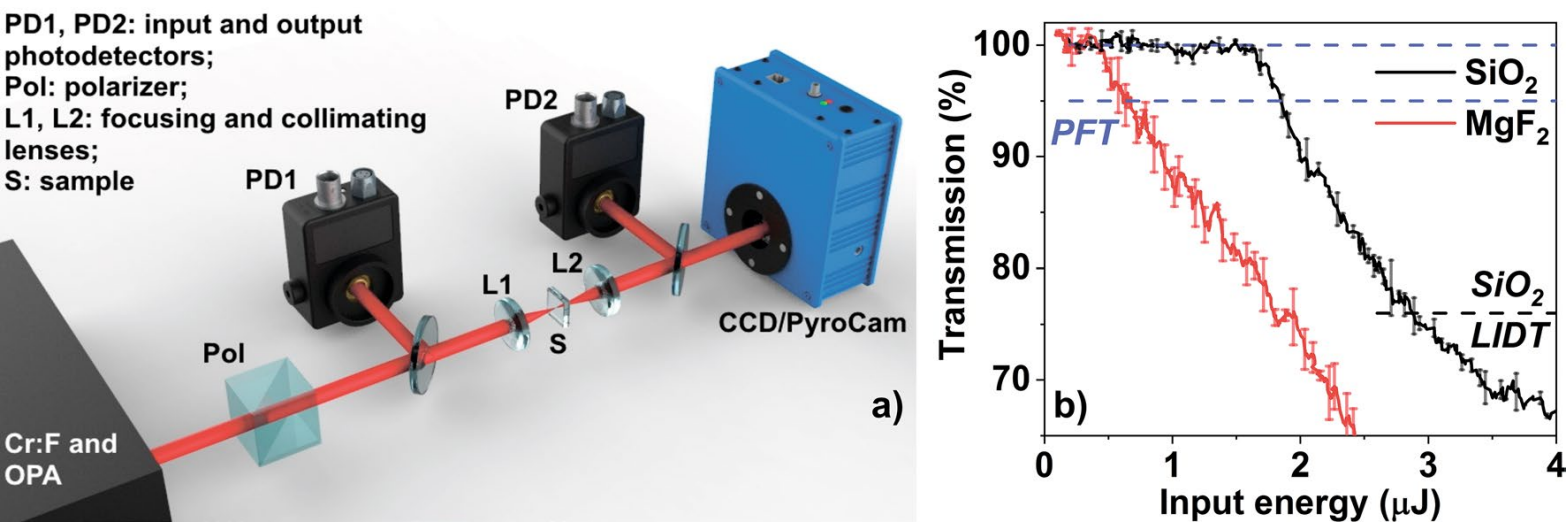
(**a**) The experimental setup: Cr:F, Cr:forsterite laser; OPA, optical parametric amplifier; L1–L2, lenses; Pol, polarizer; PD1–PD2, photodetectors; S, sample. Sample is mounted on the automated 3D translation stage. Camera (CCD or PyroCam) is moved along the optical axis to capture the beam waist. (**b**) Nonlinear transmission of the SiO$_2$ and MgF$_2$ samples measured at 2 $\upmu$m. Dashed horizontal lines represent the absence of nonlinear losses (100$\%$ transmission) and the PFT (95$\%$ transmission). As an example, the LIDT level is shown for SiO$_2$.

### Experimental set-up

The experimental set-up was based on a Cr:forsterite laser system delivering 170-fs, 2.5-mJ laser pulses centered at 1240 nm (Fig. 3). Longer wavelengths were produced using a mid-IR optical parametric amplifier (OPA). The second harmonic was generated in a 1-mm-thick type-I BBO crystal. A set of wavelengths composed of the Cr:forsterite fundamental wavelength (1240 nm), its second harmonic (620 nm), signal (1.65 $\upmu$m and 2 $\upmu$m) and idler (3.23 $\upmu$m, 3.85 $\upmu$m and 4.78 $\upmu$m) output from the OPA was selected for the experiments. The pulse duration $\tau _{FWHM}$ was characterized using a home-made second harmonic generation FROG (SHG-FROG) apparatus. We used either Thorlabs A230 ($f=4.51 {\mathrm{mm}}, NA=0.55$) or A240 ($f = 8.0 {\mathrm{mm}}, NA = 0.5$) lenses (L1 in the Fig. 3) for visible and near-IR radiation (0.62 nm–2 $\upmu$m). It is worth to report that the measured PFT does depend on the focusing conditions in our experiments similarly to^40^, nevertheless the presented dependencies of the PFT on the incident pulse wavelength have the same behaviour under two different focusing conditions described above. Mid-IR pulses (3–5 $\upmu$m) were focused by Thorlabs C036TME ($f = 4.00\,\mathrm{mm}, NA = 0.56$) lens. The typical measured beam diameter $a_{0}$ as well as the pulse duration are given for each wavelength $\lambda _0$ in the Table 1. Laser pulses were tightly focused into the samples at a depth of 0.1–0.2 mm below the surface. A Glan prism or wire grid polarizer was used to vary the incident laser pulse energy of near-IR and mid-IR radiation. The transmission of the driving pulses was measured using Si, Ge and PbSe detectors (Thorlabs Inc.). Absolute calibration was done for each wavelength using a Gentec-EO QE-B energy meter. Samples were mounted onto an automated translation stage to provide interaction of each pulse with a fresh material and thus, avoiding accumulation effects. The SiO$_2$ and MgF$_2$ samples were polished on both sides. The SiO$_2$ sample was made of a high-purity SiO$_2$. The MgF$_2$ sample was made of a single crystal cut at the (001) direction.

To determine the value of PFT, we measured the nonlinear transmission and beam profile in the focal plane for each wavelength. We varied incident energy by a polarizer and collected signal from the reference (PD1) and transmitted (PD2) photodiodes. Fig. 3 shows the transmission data for SiO$_2$ and MgF$_2$ samples at 2 $\upmu$m. The curves are normalized to take into account Fresnel losses on the boundaries. Note that the response of bounds electrons (such as generation of odd harmonics) is significantly suppressed under described experimental conditions due to high phase-mismatch. At low energies the transmission is 100$\%$ indicating the absence of linear absorption. When incident energy reaches PFT, transmission tends to decrease rapidly. The exact value of threshold is obtained when the transmission has decreased to 95$\%$, which is sufficient for confident threshold registration with 3$\%$ root-mean-square stability of the driving sources. Note that at energies several times higher than the threshold, the beam could be attenuated by the defocusing and screening effects due to high density of the microplasma. As a consequence nonlinear transmission may saturate at high energies. However, these effects lay beyond the scope of the presented paper. Beam profile was imaged onto the camera sensor by the L2 lens, which was the same as L1. We used a silicon CCD in the case of 0.62 $\upmu$m and 1.24 $\upmu$m wavelengths and Spiricon Pyrocam IV in the case of other wavelengths. The camera was placed at such a distance to reach 10x-20x and 30x-50x (depending on wavelength) beam magnification in the case of silicon and pyroelectric sensors, respectively. Since beam profile stays almost unaffected by the propagation due to tight focusing and low input energy, we used fluence measured at the focal plane to characterize the PFT. For evolution of the plasma profile with input energy one can address^34^.

The alignment of the system strongly affects the absolute value of the PFT. Therefore nonlinear transmission and beam diameter were measured several times (5–6) for each wavelength to evaluate the statistical mean value of the measurements. This mean value is shown in Figs. 1 and 2 as square symbols for each wavelength, while the error bars indicate standard deviation obtained as a result of averaging. Since beam diameter in the focal plane was also measured several times, Table 1 represents the mean value for each wavelength. Note that standard deviation shown in Figs. 1 and 2 originates from fluctuations of both threshold energy and beam profile, since focusing in slightly smaller diameter leads to lower threshold energy, while slightly worse focusing leads to the higher threshold energy.

**Table 1:** Typical laser pulse parameters used in the experiments.

$\lambda _0$ ($\upmu$m)	$\tau _{FWHM}$ (fs)	$a_{0}$ ($\upmu$m)
0.62	120	1.3
1.24	170	3.4
1.65	170	7.6
2.00	170	9.0
3.23	230	4.1
3.85	280	6.3
4.78	300	7.5

### Theoretical approach

MRE^31,46–49^ are used to simulate the ionization rates and the temporal evolution of the electron density in the conduction band (CB) during the laser-dielectric material interaction. The electron evolution is described by three main processes: field-induced ionization driving the electron transitions from the valence band (VB) to the bottom of the conduction (CB), field-induced electron transitions from each CB level to the closest upper CB level (corresponding to one-photon absorption in the CB), impact ionization bridging the electrons of the highest CB level to the VB, both then being transferred to the bottom of the CB. The electron recombination is neglected since it is expected to play a role on a timescale longer than the pulse duration. Indeed, for MgF$_2$, the recombination timescale lies in the picosecond range^43,50^. For SiO$_2$, the exciton formation exhibits a characteristic time close to the pulse duration^51^. The absence of the recombination process in the modeling may lead to an underestimation of the PFT but will not change the overall behaviour of the PFT dependence on the laser pulse wavelength.

We consider one VB level and a finite number *N* of allowed energy levels in the CB. The corresponding indexes for the VB and CB levels are $i = 0$ and $0 < i \le N$, respectively. The levels in the CB have been chosen to have the following energies:

1 $$\begin{aligned} E_i = E_g + (i-1)\hbar \omega _0, \end{aligned}$$

where $\hbar \omega _0$ is the photon energy of the incident light and $E_g$ is the bandgap energy of the considered material. The highest considered CB level $i=N$ must have an energy $E_N-E_1$ that is sufficiently large to fulfill energy and momentum conservation during the impact ionization process:

2 $$\begin{aligned} E_N - E_1 \ge 1.5 \, E_g, \end{aligned}$$

where the effective electron mass was set to 0.5m$_e$^31^. From Eqs. (1) and (2), the number of CB levels is:

3 $$\begin{aligned} N = 1 + \left\lfloor \frac{ 1.5 \, E_g}{\hbar \omega _0} \right\rfloor , \end{aligned}$$

where $\lfloor x \rfloor$ is the floor function (maximum integer that is less or equal than *x*). This estimation follows from Eq. (8) in^46^ assuming that the electron masses in VB and CB are both equal to the free-electron mass and neglect the mean oscillation energy of the applied electric field. For instance, when the photon energy of the incident light is equal to $\hbar \omega _0=3$ eV and the band gap is $E_g=9$ eV, we have to consider $N=6$ energy levels in the CB according to Eq. (3).

The equation governing the laser-induced electron dynamics in the considered VB and CB levels reads as follows:

4 $$\begin{aligned} \left\{ \begin{array}{l} \partial _t{\rho _{0}(t)} = -W_{PI}\rho _{0}/n_{at}-\nu _{imp}\rho _{N} \\ \\ \partial _t{\rho _{1}(t)} = \ \ W_{PI}\rho _{0}/n_{at}- \nu _{1ph}\rho _{1} + 2\nu _{imp}\rho _{N} \\ \\ \partial _t{\rho _{i}(t)} = \ \ \nu _{1ph}\rho _{i-1}-\nu _{1ph}\rho _{i} \ for \ 1<i<N \\ \\ \displaystyle \partial _t{\rho _{N}(t)} = \ \ \nu _{1ph}\rho _{N-1} -\nu _{imp}\rho _{N} \end{array} \right. \end{aligned}$$

where $\rho _{j}$ is the population of the corresponding energy level *j*, $n_{at}$ is the density of neutrals, $W_{PI}$, $\nu _{1ph}$ and $\nu _{imp}$ are the photoionization rate, one-photon electron transition frequency in the CB and impact ionization frequency, respectively.

The photoionization rate $W_{PI}$ is described by the well-known Keldysh model^25^ which includes both multiphoton absorption and tunneling. The band gap of SiO$_2$ and MgF$_2$ are taken equal to 9 eV^52^ and 10.8eV^29^, respectively. The above threshold ionization is not included in the present approach since its contribution is expected to be negligible.

To define the electron transition frequency $\nu _{1ph}$ in the CB, we assume applicability of the weak-field approximation^26,45^ and account only for processes in which one photon is involved. Thus, the electron transition frequencies are due to two main mechanisms: one-photon direct transitions and indirect phonon-assisted transitions. The one-photon direct transitions of electrons in the CB, $W_{1ph}^{dr}$, is proportional to $\omega _0^{-2}$, where $\omega _0$ is the laser frequency^53^. The indirect process corresponding to phonon-assisted one-photon absorption, $W_{1ph}^{idr}$, is proportional to $\omega _0^{-4}$^45,53,54^. For the levels in the CB, the direct and indirect one-photon absorption processes are introduced as follows:

5 $$\begin{aligned} \nu _{1ph}\ = \left( W_{1ph}^{dr}+W_{1ph}^{idr}\right) E_0(t)^2, \end{aligned}$$

where $E_0$ is the time dependent envelope of the electric field in the glass sample. To model the laser-induced ionization process in SiO$_2$ and MgF$_2$, we set the rates for a photon energy of $\hbar \omega$ = 1.5 eV as follows: $W_{1ph}^{dr}=3.6\times 10^{-7}\, \mathrm{m}^2\mathrm{V}^{-2}\mathrm{s}^{-1}$, $W_{1ph}^{idr} = 4\times 10^{-8}\,\mathrm{m}^2\mathrm{V}^{-2}\mathrm{s}^{-1}$ and $W_{1ph}^{dr} = 1\times 10^{-6}\,\mathrm{m}^2\mathrm{V}^{-2}\mathrm{s}^{-1}$, $W_{1ph}^{idr} = 2.8\times 10^{-7} \mathrm{m}^2\mathrm{V}^{-2}\mathrm{s}^{-1}$, respectively. These values for one-photon absorption rates in CB are close to the one used in^47^ and allow us to reproduce the experimental observations. Slight variations of these parameters lead to a non-significant variations of the presented results, and the predicted trends and conclusions remain the same.

In case of MgF$_2$, we operate with wavelengths above 2 $\upmu$m, at which the laser pulse intensities required for PFT reach the strong field regime^26^ and the multiphoton interactions in the CB is not negligible^55^. To account for this fact, we calculate the following parameter that indicates the transition from quantum to classical regime of laser-matter interaction ^26^:

6 $$\begin{aligned} s = \frac{2e^2E_0(t)^2}{m^{*}\omega _0^2} / \hbar \omega _0 \end{aligned}$$

If *s* exceeds a threshold value s$^{sf}$ significantly larger than unity, the transition to the classical interaction regime is fully reached, and the strong field approximation is applied to calculate the effective transition rate of the electron transitions between the neighbouring CB levels. We set s$^{sf}$ = 400, providing a PFT close to experimental observations as shown below. Within the strong-field approximation, the effective electron transition rate, $\widetilde{W}_{1ph}^{sf}$, no longer exhibits a dependence on the photon energy^26^. The field dependence also changes, becoming inverse proportional to the laser electric field amplitude $E_0(t)$^26^. If the strong-field condition for the parameter *s* is reached, for each level in the CB, the absorption rate is evaluated as follows:

7 $$\begin{aligned} \nu _{1ph}\ = \widetilde{W}_{1ph}^{sf}/E_0(t), \end{aligned}$$

where the $\widetilde{W}_{1ph}^{sf}$ value is chosen in order to fulfill the continuity of the $\nu _{1ph}$ with the electric field amplitude $E_0(t)$ varying from the low values where the weak field approximation is applicable up to the larger values where the strong field approximation for $\nu _{1ph}$ calculation is applied. Values of the electric field amplitude $E_0^{sf}$ and intensity $I_0^{sf}$ corresponding to the indicated threshold s$^{sf}$ are given in the Table 2 together with the $\widetilde{W}_{1ph}^{sf}$ values in MgF$_2$ for the set of the experimental wavelengths. For wavelengths shorter than 2 $\upmu$m, the threshold electric field amplitude $E_0^{sf}$ and intensity $I_0^{sf}$ are well above the PFT values and thus the strong field approximation is not applicable for CB transitions description. For wavelengths longer than 2 $\upmu$m, the electric field amplitude $E_0^{sf}$ and intensity $I_0^{sf}$ are below the values corresponding to the PFT, and thus the strong field approximation for CB transition rate is applied.

**Table 2:** Electric field amplitude $E_{0}^{sf}$ and intensity values $I_0^{sf}$ indicating strong field regime and effective electron transition rate $\widetilde{W}_{1ph}^{sf}$ in strong field regime for CB transitions in MgF$_2$ at experimental wavelengths.

$\lambda _0$ ($\upmu$m)	$E_{0}^{sf}$ (V m^−1^)	$I_0^{sf}$ (W cm$^{-2}$)	$\widetilde{W}_{1ph}^{sf}$ (m^−1^ V s^−1^)
0.62	1.0 $\times 10^{11}$	1.9 $\times 10^{15}$	7.0 $\times 10^{26}$
1.24	3.6 $\times 10^{10}$	2.4 $\times 10^{14}$	1.7 $\times 10^{26}$
1.65	2.4 $\times 10^{10}$	1.0 $\times 10^{14}$	1.1 $\times 10^{26}$
2.00	1.8 $\times 10^{10}$	5.7 $\times 10^{13}$	8.5 $\times 10^{25}$
3.23	8.6 $\times 10^{9}$	1.3 $\times 10^{13}$	5.1 $\times 10^{25}$
3.85	6.6 $\times 10^{9}$	7.9 $\times 10^{12}$	4.5 $\times 10^{25}$
4.78	4.8 $\times 10^{9}$	4.1 $\times 10^{12}$	3.8 $\times 10^{25}$

The impact ionization frequency is set to $\nu _{imp} = 1/\tau _{imp}$, where $\tau _{\mathrm{imp}}$ is the characteristic impact-ionization timescale. The characteristic timescale of impact ionization for considered materials is set to $\tau _{\mathrm{imp}} \sim 1$ fs^56^.

The electric laser field is defined in the time interval $0 \le t \le \tau _0$ by

8 $$\begin{aligned} E_0(t) = \mathcal{E}_0 \sin ^2\left( \pi t/\tau _0\right) \end{aligned}$$

where $\omega _0$, $\tau _0$ and $\mathcal{E}_0$ are the central angular frequency, the total duration and the amplitude of the laser pulse, respectively. The pulse duration defined at the FWHM (full width at half maximum) is calculated as follows:

9 $$\begin{aligned} \tau _{FWHM} = 0.3641 \tau _0. \end{aligned}$$

The laser intensity is defined as $I_0 = n_0 \varepsilon _0 c \, \mathcal{E}_0^2 /2$, where *c* is the speed of light, $\varepsilon _0$ is the vacuum permittivity and $n_0$ is the refractive index of the glass sample^57,58^. We consider laser pulses with $\tau _{FWHM}$ equal to the experimentally used values, photon energy $\hbar \omega _0$ varying from 0.25 to 2 eV (i.e., from the mid-infrared to the visible spectral regions), and a peak laser-pulse intensity $I_0$ ranging from $10^{9}$ up to $5\times 10^{14}$ W/cm$^{2}$.

The total CB electron density, $\rho _e (t)$, is evaluated as follows:

10 $$\begin{aligned} \rho _{\mathrm{e}}(t) = \, \sum _{i>0}^N \rho _{i}(t) \,\text{. } \end{aligned}$$

The initial conditions are $$\rho _0(t=0) = n_{at}$$, in case of SiO$$_2$$$$n_{at} = 2.2\times 10^{22} cm^{-3}$$ and $$n_{at} = 6.5 \times 10^{22} cm^{-3}$$ in case of MgF$$_2$$. The CB levels are empty: $$\rho _{i>0}(t=0) = 0$$.
